# Phylogeography and Genetic Differentiation among Populations of the Moon Turban Snail *Lunella granulata* Gmelin, 1791 (Gastropoda: Turbinidae)

**DOI:** 10.3390/ijms14059062

**Published:** 2013-04-25

**Authors:** Yuh-Wen Chiu, Hor Bor, Mian-Shin Tan, Hung-Du Lin, Chuen-Tan Jean

**Affiliations:** 1 National Museum of Marine Biology and Aquarium, Checheng, Pingtung 944, Taiwan; E-Mail: chiuyw@nmmba.gov.tw; 2 Department of Biomedical Science and Environmental Biology, Kaohsiung Medical University, Ksohsiung 807, Taiwan; E-Mails: emilebor@msn.com (H.B.); m795027@kmu.edu.tw (M.-S.T.); 3 Department of Physical Therapy, Shu Zen College of Medicine and Management, Kaohsiung 821, Taiwan; E-Mail: varicorhinus@hotmail.com; 4 The Affiliated School of National Tainan First Senior High School, Tainan 701, Taiwan

**Keywords:** *Lunella granulata*, mainland China, MtDNA, phylogeography, Taiwan

## Abstract

We examined the genetic variation and phylogeographic relationships among 10 populations of *Lunella granulata* from mainland China, Penghu Archipelago, Taiwan Island, and Japan using mitochondrial COI and 16S markers. A total of 45 haplotypes were obtained in 112 specimens, and relatively high levels of haplotype diversity (*h* = 0.903) and low levels of nucleotide diversity (π = 0.0046) were detected. Four major phylogenetic lineage clusters were revealed and were concordant with their geographic distribution, agreeing with the haplotype network. These results suggested that geographic barrier isolating effects were occurring among the populations. This hypothesis was also supported by a significant genetic differentiation index (*F*_ST_ = 0.709) and by a spatial analysis of molecular variance (SAMOVA) analysis. A mismatch distribution analysis, neutrality tests and Bayesian skyline plots found a single significant population expansion. This expansion occurred on the coast of mainland China before 20–17 ka. Consequently, although the dispersal ability of the planktonic stage and the circulation of ocean currents generally promote genetic exchanges among populations, *L. granulata* has tended to maintain distinct genetic groups that reflect the respective geographic origins of the constituent lineages. Although the circulation of ocean currents, in principle, may still play a role in determining the genetic composition of populations, long-distance migration between regions is difficult even at the planktonic stage.

## 1. Introduction

Information on population structure is required for management and conservation strategy design in marine species facing overexploitation and extinction risk [1]. Based on the high resolution uniquely imbedded in nucleotide composition, the use of molecular markers and other genetic tools to effectively reveal genetic dynamics provides a means of testing how target species, either wild or captive, react to environmental stimuli. To counteract the significant decline in fisheries resources, an understanding of population genetic structure and the distribution of genetic diversity among populations is needed for conservation management of economically important species [2].

The moon turban snail (*Lunella granulata* Gmelin, 1791), distributed primarily in the Indo-West Pacific area and ranging from Japan and Korea to China, preferentially inhabits rocky shores in the intertidal zone [3–5]. From an economic perspective, *L. granulata* is one of the most important species for the residents of the Penghu Archipelago and Taiwan due to its abundance and widespread distribution. The life history of the moon turban snail begins with the planktonic larval stage, which begins one day after hatching. The congener *L. coreensis* maintains its planktonic larval stage for 2.5 days [4–6]. During the planktonic larval period, potential geographic barriers hampering the movement of individuals among geographically distinct populations would generally be less effective. As a result, the combination of a planktonic larval period and ocean current patterns could play a major role in determining individual migration and genetic variation among *L. granulata* populations.

The inherent dispersal ability of species and the lack of significant oceanic geographic barriers result in high levels of gene flow among marine populations, reducing the potential for detectable genetic structure over a large geographic scale [7]. Previous studies of a subtidal gastropod with a short planktonic stage, the Japanese turban shell *Turbo* (*Batillus*) *cornutus*, have revealed a clear genetic structure that corresponds to the pattern of warm currents along the Japanese coastline [8,9]. Several studies have found clear genetic differentiation among populations of other widespread marine species [10,11].

It has been proposed that the occurrence of a pelagic larval stage in benthic marine species and the dispersal capacity of the larvae act jointly to shape genetic structure. This hypothesis has been examined in marine gastropod species such as *Littorina* spp. [12] and abalone (*Haliotis cracherodii*) [13] and in other invertebrates with a planktonic larval stage [14–16]. Moreover, the diverse life history within each species includes the niche and habitat, the dynamic circulation of regional ocean currents and past historical events. These factors may also contribute to the formation of distinct genetic groups [17]. However, no direct relationship was found between the direction of ocean currents and genetic structure in representative marine species despite the inherently high dispersal ability of these species [18].

The development of molecular techniques and analytical tools has provided high-resolution genetic information and the potential to determine the genetic structure of populations. These tools include the polymerase chain reaction (PCR), sequencing of DNA fragments, and phylogenetic analysis of allelic variants. Given the broad distribution of *L. granulata* in southeast Asia, the aim of this study is to examine the population genetic structure and phylogeographic patterns of *L. granulata* throughout mainland China and adjacent islands, including the Penghu Archipelago and Taiwan, based on concatenated mtDNA nucleotide sequences from COI and 16S genes. This region is also characterized by the complex circulation patterns of ocean currents as well as the occurrence of the last glacial maximum (LGM) approximately 10,000 years ago. A hierarchical scale-based analysis of molecular variation and descriptive genetic information would be helpful to define the evolutionary history of *L. granulata* and to provide evidence about the effects of environmental dynamics on intra-species genetic structure.

## 2. Results and Discussion

### 2.1. Results

#### 2.1.1. Genetic Diversity of *L. granulata*

**Figure 1:**
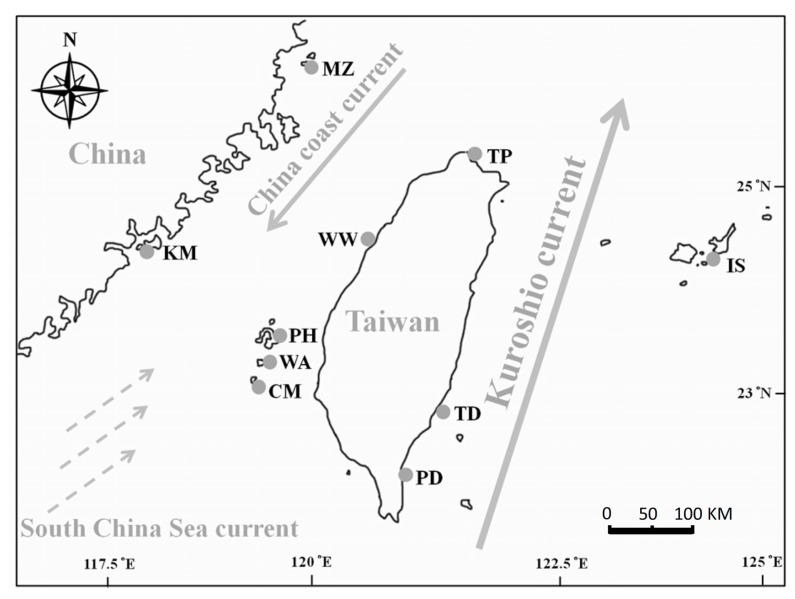
Map of the distribution of *L. granulata* and sampling locations in the present study.

Specimens of *L. granulata* were selected from 10 populations including mainland China, Taiwan, and Japan (Table 1; Figure 1). The combined 1244-bp fragment of the mtDNA COI and 16S genes contained 48 polymorphic sites, 21 singleton substitutions and 27 parsimony informative sites, and 30 haplotypes were generated (Table 1). The sequences have been deposited in GenBank under accession numbers 16S: KC535558–KC535669 and COI: KC535670–KC535781. The A/T base content was higher than the C/G base content in the sequences examined (mean: A = 27.7%, T = 36.0%, C = 16.1%, and G = 20.2%). This result is consistent with previous studies showing that the COI and 16S genes tend to be an A-T-rich region of the mitochondrial genome [19,20]. The mean haplotype diversity was high (*h* = 0.899), with values ranging from 0.154 in Ishigaki (IS) to 0.933 in Wangan (WA). The samples from Japan (IS) presented the lowest nucleotide diversity (π = 0.0001) (Table 1), similar to that found for Taiwan (PD, TD and MZ) (π = 0.0004–0.0005). In contrast, the Miaoli (WW) samples showed a higher nucleotide diversity (π = 0.0035), comparable to Wangan (π = 0.0026) (Table 1).

#### 2.1.2. Phylogenetic Analysis

**Figure 2:**
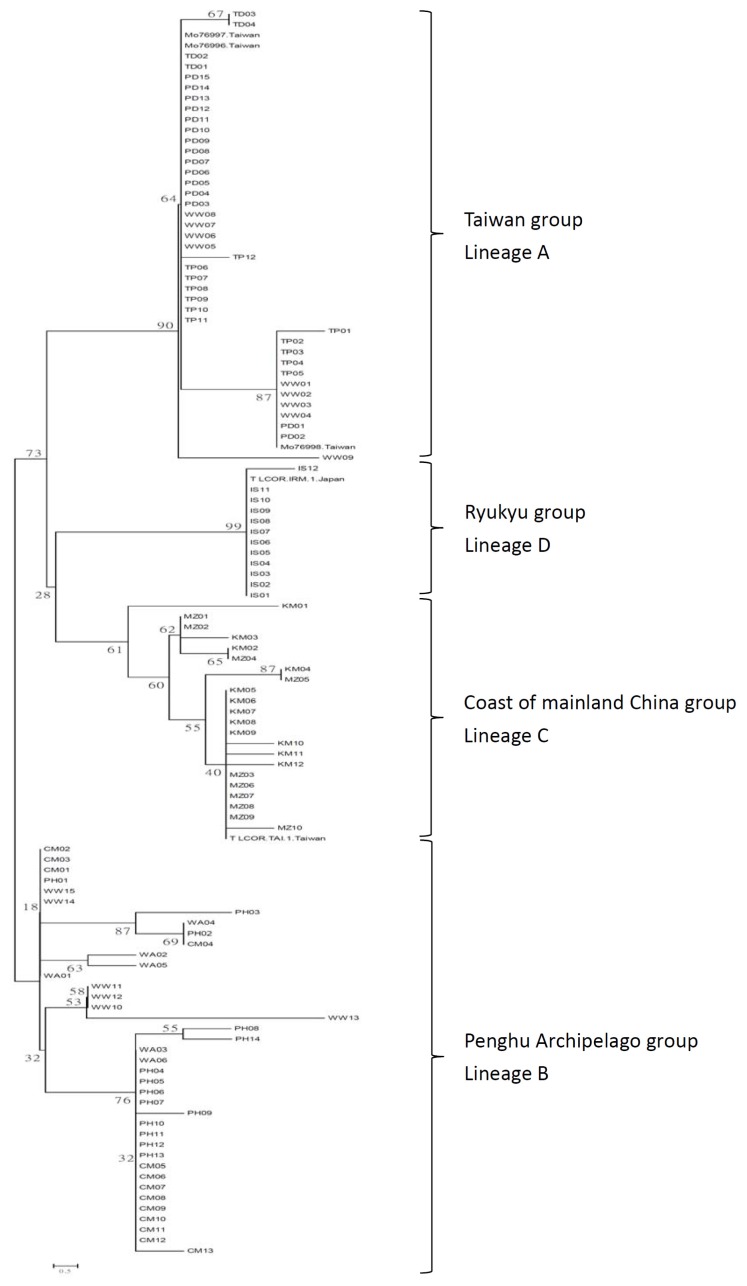
Neighbor-joining tree of individual sequences of mtDNA COI and 16S region genes in *Lunella granulata*. Numbers at the nodes indicate bootstrap values (expressed as percentages) with 1000 replicates.

We conducted a phylogenetic analysis based on the combined nucleotide sequences of the COI and 16S genes. A neighbor-joining (NJ) tree reconstructed from the concatenated nucleotide sequences (COI-16S tree) is shown in Figure 2. Ten populations formed four distinct lineages in relation to the geographic distribution. Lineage A comprised seven haplotypes in 42 individuals from four populations sampled from Taiwan Island, and Lineage B contained ten haplotypes in 33 individuals specific to Penghu Archipelago and three haplotypes in six individuals belonging to the Miaoli population (Lineage A). Lineage C primarily included populations from the coast of mainland China (Kinmen and Matsu). Only two haplotypes in 13 individuals belonged to Lineage D sampled from Japan (Figure 2).

**Figure 3:**
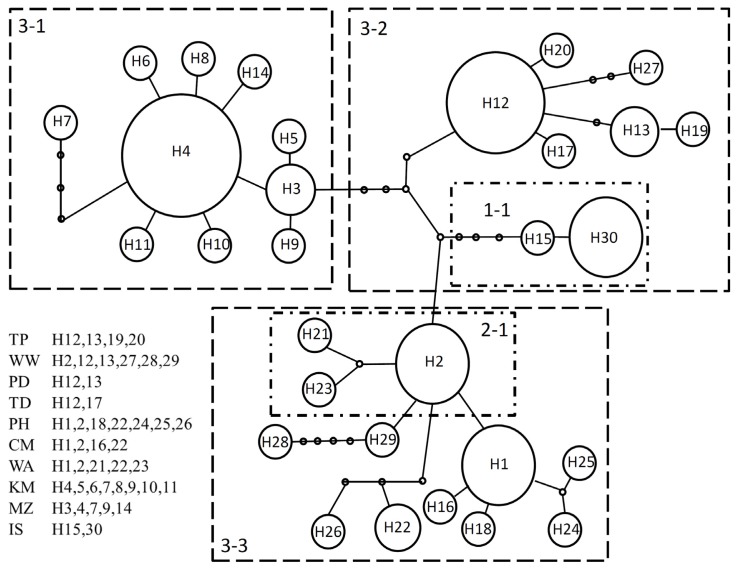
Minimum spanning network based on mutations between haplotypes observed in 10 populations of *L. granulata*.

Based on a network analysis, the observed mtDNA haplotypes were separated into one 1-step clade (1-1) and one 2-step clade (2-1); the last group included all haplotypes in three 3-step clades (3-1, 3-2 and 3-3) (Figure 3). The major distribution of 3-step clades revealed that clade 3-1 was composed of the Kinmen and Matsu populations (Lineage C), clade 3-2 contained most of the Taiwan and Ryukyu populations (Lineage A) and clade 3-3 included the Penghu Archipelago populations and the WW population (Lineage B). The haplotypes within subclade 1-1 were all from Ryukyu and originated from Penghu Archipelago and Miaoli within subclade 2-1.

#### 2.1.3. Historical Demography

**Figure 4:**
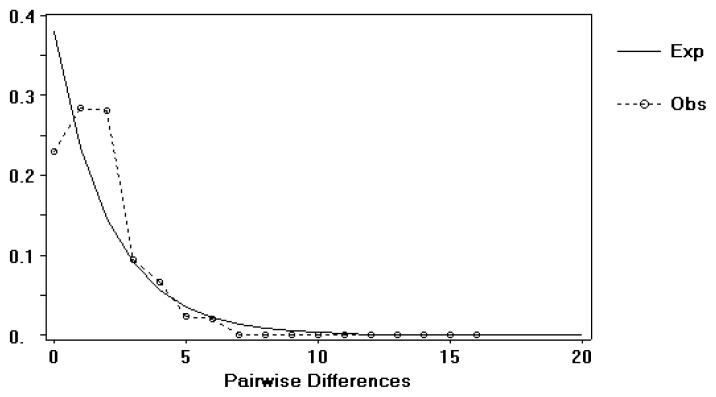
Mismatch-distribution analysis of *L. granulata* mtDNA haplotype sequences in lineage C. A simulated Poisson distribution is indicated by a dotted line.

The observed moderate to high haplotype diversity coupled with a low nucleotide diversity in all lineages (Table 1) indicated the possibility of rapid population growth [21,22]. For populations from the coast of mainland China (lineage C), the negative values of both Tajima’s *D* and Fu’s *Fs* tests supported demographic expansion, with the latter test showing particularly clear statistical significance [23]. In addition, the same scenario was also supported by alternative methodology, such as a mismatch distribution analysis (Figure 4) and an examination of the allele frequency distribution under a sudden expansion model (Table 1, SSD test, *p* > 0.05). In terms of the beginning of population expansion, the τ value of 1.156 derived by Arlequin suggested a time approximately 19,000–66,000 years before the present, occurring during the last glacial maximum (LGM) in the Pleistocene. For the populations in Taiwan and Penghu Archipelago, the non-significant value of both Tajima’s D and Fu’s *Fs* values obtained from intra-populational and intra-regional data implied a scenario based on stable population demographics. Interestingly, except for the populations from the coast of China, the analysis of the mismatch distribution for the total populations did not identify any significant deviations from the expected distribution of differences under a stepwise expansion model (most *p*-values > 0.05 based on the sum of squared distances).

**Figure 5:**
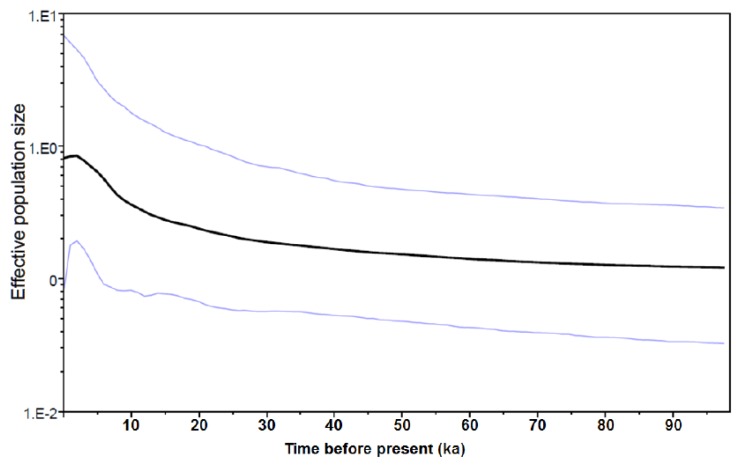
Bayesian skyline plot (BSP) of the effective population size through time for *L. granulata* in lineage C.

A Bayesian skyline plot (BSP) analysis was used to date the shifts in population size of the *L. granulata* lineages. The results revealed stable population sizes through time for lineages A and B, but a pattern of population growth occurring during 20–17 ka was detected in lineage C (Figure 5).

#### 2.1.4. Population Differentiation

The existence of phylogeographic structure was examined following Pons and Petit [24] by calculating two genetic differentiation indices, *N*_ST_ and *G*_ST_. A comparison of *N*_ST_ and *G*_ST_ revealed a strong correlation between phylogeny and geography, with *N*_ST_ substantially greater than *G*_ST_ (0.709 and 0.305, respectively). The overall differentiation among populations was very high (*F*_ST_ = 0.70). An AMOVA revealed a high degree of structuring, with the largest proportion of the variation (71.31%; *p* < 0.0001) among geographic groups and significantly lower proportions of variation among populations within geographic district (3.49% of total variation) and within populations (25.2% of total variation) (Table 2). The number of groups with the highest Φ_CT_ was four (*K* = 4) based on a spatial analysis of molecular variance (SAMOVA) (Table 3). This configuration was consistent with the lineage clusters depicted in the NJ tree (Figure 2). These four groups were as follows: (1) TP, WW, PD and TD (Taiwan Island group); (2) PH, CM and WA (Penghu Archipelago group); (3) KM and MZ (coast of mainland China group) and (4) IS (Ryukyu group). A Mantel test for *L. granulata* indicated a weak but significant correlation between pairwise population *F*_ST_ values and geographic distances (*r* = 0.00038, *p* < 0.001). The θ values from MIGRATE for each lineage showed that Penghu Archipelago maintained the largest effective population size, whereas the smallest effective population size was found for Taiwan Island (Table 4).

**Figure 6:**
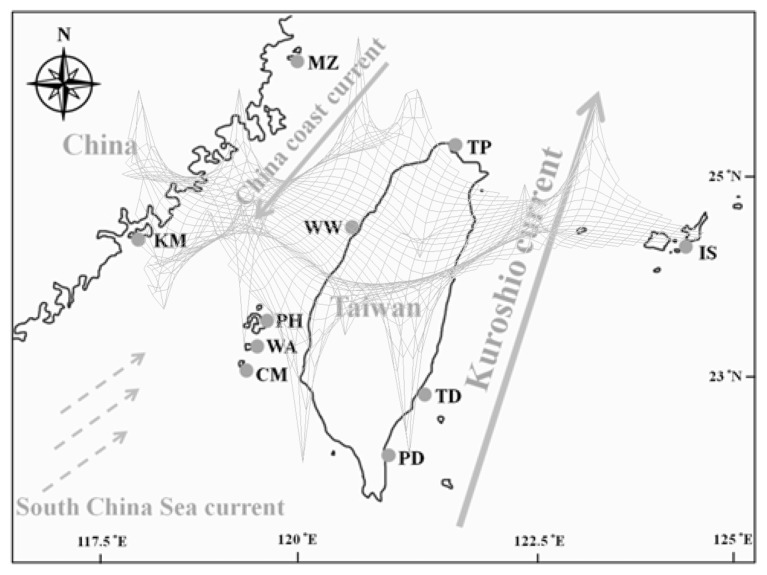
“Alleles in Space” interpolation plot for mtDNA COI and 16S region genes. UTM (northing) is on the *y* axis, UTM (easting) is on the *x* axis and residual genetic distance (Nei’s unbiased D) on the *z* axis. Positive “peaks” represent high genetic discontinuities, and negative peaks represent high genetic similarities.

The surface plots produced from the genetic landscape interpolation with different distance weighting parameters were qualitatively similar to one another. Overall, there was a strong pattern of high genetic differentiation (indicated by peaks) among the Penghu Archipelago samples (Figure 6).

### 2.2. Discussion

#### 2.2.1. Historical Demography and Genetic Diversity

Nucleotide and haplotype diversities can provide demographic information on the history of *L. granulata* populations. Grant and Bowen [21] introduced four basic scenarios for population demographic history by comparing haplotype and nucleotide diversity. In this study, high haplotype diversity and low nucleotide diversity were observed in the COI and 16S region for all populations examined. This pattern could be attributed to a recent population expansion after a low effective population size caused by either founder events or bottlenecks [21,25]. Moreover, we found that the genetic diversity of *L. granulata* is much lower than that of other marine gastropod species (*Cittarium pica*, COI, π = 0.015; 16S, π = 0.017 [26] and *Rapana venosa*, COI + 16S, π = 0.0041–0.0067 [27]). Although diverse anthropogenic and ecological factors potentially determine genetic diversity, the low genetic diversity of *L. granulata* could be due primarily to two factors, overexploitation [28] and a shore-specific environmental factor [29]. The high genetic diversity of marine organisms, especially coastal fish, is explained by the tendency to maintain a stable population size due to inherently limited dispersal ability, thus producing decreased gene flow among discontinuous populations. Accordingly, the occurrence of exclusive haplotypes within *L. granulata* lineages reflects the limited amount of migration occurring on ocean currents (Table 4). The network analysis results placed the Ryukyu group at the tip of the diagram. The genetic diversity of this group was lower than that of any other population analyzed. We suggest that the recolonization effect on the Ryukyu Islands associated with the glacial retreat would result in a signature corresponding to reduced genetic diversity.

#### 2.2.2. Phylogeographic Patterns

In this study, the phylogenetic reconstruction performed for *L. granulata* identified genetic groups that partially corresponded to geographic origins. This result suggested the dominant effects of geographic isolation due to the lower sea level in Taiwan Strait during the Pleistocene era, preventing gene flow among Taiwan Island, Penghu Archipelago and mainland China. The phylogeographic signal based on the presence/absence of haplotypes in the MSN (Figure 3) revealed information about the origin and history of *L. granulata* populations. The haplotype network indicated that the haplotypes in subclade 2-1, which originated primarily from Penghu Archipelago, were the ancestral haplotypes because they were located at internal nodes in the network. Interestingly, the clade 3-1 haplotypes from mainland China coast and clade 3-2 haplotypes in Taiwan and Japan tended to be placed at terminal or external positions in the network, suggesting a recently derived origin. According to the network, there were at least two major migratory routes for *L. granulata*, one from the Penghu Archipelago (clade 3-3) to the mainland China coast (clade 3-1), and the other from the Penghu Archipelago through Taiwan (clade 3-2) to Japan. The MIGRATE analysis also supported that gene flow occurred only from Penghu Archipelago (clade 3-3) to Taiwan (clade 3-2) and Japan (clade 1-1).

The distribution of marine fauna along the coast of southern China is primarily driven by the circulatory pattern of complex ocean currents [30,31]. During summer, the South China Sea Warm Current flows northwards from the South China Sea towards the East China Sea [32]. In addition, one branch of the Kuroshio Current, originating from the equator, flows northwards along the eastern coast of the Philippines and Taiwan [33]. In winter, a branch of the northwards China Coastal Current could be deflected by the northeast monsoon to flow back into Taiwan Strait [34] but would be blocked at Penghu Island by the circulation of deeper waters, which creates a barrier against northwards flow [35] and prevents the flow of the China Coastal Current in Taiwan Strait in winter.

#### 2.2.3. Population Genetic Structure and Historical Demography

In contrast to terrestrial species, marine organisms are usually expected to show little genetic differentiation due to the absence of geographic barriers [36]. Generally, most marine species show low levels of genetic differentiation among geographic regions [37]. This outcome is most likely due to the high dispersal capability of the planktonic stage or to juvenile or adult migrations between ocean basins or adjacent continental margins [17,38]. To test for population subdivision, different combinations of distinct populations were evaluated according to their geographic distribution. According to a SAMOVA, the largest difference between groups of populations (71.31%) was found if the populations were divided into four clusters. Consequently, isolation due to geographic barriers appeared to be the major determinant of the current population genetic structure of *L. granulata*. The dominant geographic structure detected in *L. granulata* indicates limited gene flow among lineages, most likely reflecting limited dispersal ability during the early life history. Otherwise, most lineages also harbored “unique” haplotypes, indicating a very low connectivity among geographic groups. A Mantel test detected a weak but significant correlation between genetic dissimilarity and geographic distance, indicating that the isolation by distance pattern might not be the driving force for the differentiation of the lineages. For *L. granulata*, the results suggested a good agreement between hydrographic barriers and the genetic differentiation of geographically distinct populations. In addition, a clear pattern of both demographic and range expansion was observed along the coast of mainland China. The beginning of rapid population growth at 17,000–20,000 bp indicated that the most recent expansion occurred during the interglacial period prior to the LGM, 23,000–18,000 bp [39], during which sea levels decreased 120–140 m [32]. A total land area estimated at 850,000 km^2^ was exposed on the East China Sea Shelf during the Pleistocene ice ages, and the contraction and expansion of the available land masses are thought to have greatly influenced the distribution of species, including that of *L. granulata* (reviewed [40]). Therefore, based on all aspects of the analyses in this study, we suggest that the extreme climatic conditions during the Pleistocene period, with the alternation of glaciations and interglacial periods, had a strong influence on the contemporary distribution of the genetic variation of *L. granulata*, as often reported in other marine species [41].

## 3. Experimental Section

### 3.1. Sampling

A total of 112 snails were collected from four regions including mainland China, Taiwan and Japan. These samples included 45 individuals from four sites (Shimen, Wanwa, Gangzai and Shanyuan) in Taiwan; 33 samples from three sites (Panghu, Qimei and Wang-an) in Panghu Archipelago; 22 individuals from two sites (Kinmen and Matsu) on the coast of mainland China; and 12 samples from Ishigaki, Ryukyu, Japan (Figure 1). For two of the mtDNA segments, we also added five published sequences of *L. granulata* (accession numbers COI: AB588888, AB588889, AB588890, FR693999 and FR694004; 16S: AB588856, AB588857, AB588858, FR694551 and FR694556). A small piece of foot tissue was cut off from each specimen. The tissue samples were then preserved in 95% ethanol and stored at 4 °C.

### 3.2. DNA Extraction, Amplification, and Sequencing

We extracted genomic DNA with a High Pure PCR Template Preparation Kit (Roche) from approximately 10 mg of tissue sample. The mitochondrial COI and 16S genes were used as molecular markers in this study. A fragment of COI (658 bp) was amplified by primers LCO 1490: 5′-GGTCAACAAATCATAAAGATATTGG-3′ and HCO 2198: 5′-TAAACTTAGGGTGACC AAAAATCA-3′ [42], and a 16S fragment (approximately 586 bp) was amplified by the primers 16Sar: 5′-CGCCTGTTTATCAAA AACAT-3′ and 16Sbr: 5′-CCGGTCTGAACTCAGATCACGT-3′ [43]. The PCR amplifications were performed in a Thermalcycler 9700 (Applied Biosystems, Foster City, CA, USA) under the following conditions: initial 3-min denaturation at 90 °C, 35 alternating cycles of 30 s at 90 °C for denaturation, 15 s at 50 °C for annealing, 45 s at 72 °C for extension and a final extension at 72 °C for 7 min. Each PCR product was examined with 1% agarose gel electrophoresis to verify the amplified fragment length with a standard size marker (TaKaRa Shuzo Co., Kyoto, Japan). The PCR products were purified with a QIA quick PCR Purification Kit (QIAGEN, Valencia, CA, USA). Both strands were sequenced using a BigDye Terminator Cycle Sequencing Kit (version 2.0, PE Biosystems, Foster City, CA, USA) and run on an ABI Prism 3100 (Applied Biosystems) automatic sequencer according to the manufacturer’s recommendations. The primers used for sequencing were the same as those used for PCR amplification.

### 3.3. Genetic Diversity

All sequences were edited with the program DNAStar software (DNA STAR, Inc., Madison, WI, USA) and checked manually. After the sequences were aligned with MEGA 5 [44], genetic diversity was quantified at the inter- and intra-population levels with DnaSP 5.0 [45] to calculate the haplotype diversity (*h*) [46] and nucleotide diversity (π) [47]. Preliminary phylogenetic trees were reconstructed for each marker using a neighbor-joining tree with a Kimura two-parameter nucleotide substitution model, as implemented in MEGA version 5 [44]. The relative support for the tree topology was obtained by bootstrapping [48], using 1000 iterations of the data matrix. We explored hierarchical relationships at the population level using the program TCS v1.2.1 [49] to link the haplotypes into a statistical parsimony network. As an overall assessment of geographic structure affecting population differentiation, a comparison of the two fixation indices *G*_ST_ and *N*_ST_ was performed with DnaSP 5.0 [45].

### 3.4. Historical Demography

To check for deviations from neutrality, the sequence data were tested using Tajima’s *D* [50] and Fu’s *F*s test [23] in DnaSP 5.0 [45]. A significant deviation from genetic neutrality could be interpreted as a result of a recent population expansion or bottleneck or as a result of selection on the mtDNA genome [51]. To compare the observed distributions with those expected under the expansion model, we calculated the sum of squared deviations (SSD) and the Harpending’s raggedness index [52]. The timing of possible population expansions (*t*, time in generations) was calculated from the relationship τ = 2 ut [53], where τ was the mode of the mismatch distribution and u the substitution rate for the entire sequence under study. The value of *u* was calculated from *u* = μ*k*, where μ was the mutation rate per nucleotide and *k* was the number of nucleotides of the analyzed fragment.

We used a model-free coalescent-based method (the Bayesian skyline plot, [54]) implemented in the software BEAST v1.6.1 [55] to depict the change in *L. granulata* female effective population size (Nfe) since the time to the most recent common ancestor (TMRCA) of the sampled mitochondrial haplotypes. Because the mtDNA exhibited clock-like properties and we had no well-defined calibration points, we adopted a conservative approach and used a range of sequence divergence rates (0.7%–2.4% per Myr) based on data from the related family Trochidae [56–58].

### 3.5. Population Differentiation

We used a spatial analysis of molecular variance (SAMOVA 1.0, [59]) to obtain inferences about population groups. SAMOVA implements a simulated annealing approach to define groups of populations that are maximally differentiated from each other. The SAMOVA analysis was performed using 1000 initial states and geographic groupings from *K* = 2 to 7. Given an *a priori* number of groups (*K*), SAMOVA uses a simulated annealing procedure to define the group composition in which the populations within a group are as genetically homogeneous as possible (*F*_SC_ minimized) and the groups are maximally differentiated from each other (*F*_CT_ maximized). The best-fit grouping pattern was subsequently applied in an AMOVA (analysis of molecular variance) using Arlequin (version 3.5; [60]). Pairwise *F*_ST_ values were used to estimate the amount of genetic differentiation among populations. Estimates of genetic variance and differentiation were tested for significance using nonparametric permutation tests as implemented in Arlequin (version 3.5; [60]).

To determine the direction of historical gene flow, we used MIGRATE software version 3.2.6 [61], applying maximum likelihood inference. The MIGRATE analyses were conducted with a full migration model (θ and *M* were estimated jointly from the data), which was compared to a restricted model (θ was averaged and *M* was symmetrical between populations). A migration matrix model with unequal population sizes and different migration rates was assumed [61].

To test for a correlation between genetic differentiation and geographic distance, a Mantel test and a spatial autocorrelation analysis were performed using Alleles in Space [62]. This method, implemented in the program Alleles in Space [62], is based on a spatially explicit Delauney network for all sampling localities. These genetic distances were then interpolated to create a surface with peaks reflecting areas of large genetic distance and troughs representing areas of low genetic distance between nearest neighbors. The results were displayed using a 50 × 50 grid with the distance weighting parameter set at values ranging from 0.1 to 2.

## 4. Conclusions

Our study shows that the populations of *L. granulata* that we analyzed exhibit a strong genetic structure corresponding to their geographic origins. *L. granulata* was divided into four population groups by SAMOVA. This result was consistent with the lineage clusters (Taiwan Island group, Penghu Archipelago group, coast of mainland China group, and Ryukyu group) depicted in the NJ tree. As a consequence, the dominant geographic structure in *L. granulata* indicated a limited level of gene flow among geographic lineages, suggesting that geographic distance, rather than a mobile planktonic stage and circulating ocean currents, was the factor that determined the genetic distribution. Interestingly, the demographic pattern based on BSP and mismatch analysis revealed a population expansion only on the coast of mainland China during the glacial retreat, in contrast with the results of other studies conducted in the same region. As a result, *L. granulata* tends to maintain a stable population structure without suffering from the dynamic effects of population size and glacial change. The distinct geographic groups identified by the analysis were a result of the inefficiency of dispersal at the planktonic stage and the limited ability of circulatory ocean currents to drive genetic exchanges among populations of *L. granulata*.

## Figures and Tables

**Table 1:** Genetic diversity statistics and tests of selective neutrality for *Lunella granulata* populations.

Regions	Population	Site code	Coordinates	*N*	*N*h		*h*	π (10^−2^)	Tajima’s D	Fu’s *Fs*	*R*_2_	SSD
Taiwan Island (Lineage A)				48	9		0.659	0.18	−1.246	−0.694	0.069	0.538 *
	Taipei	TP	25.29°N 121.57°E	12	4	H12,13,19,20	0.682	0.11	0.184	0.097	0.162	0.040
	Miaoli	WW	24.60°N 120.73°E	15	6	H2,12,13,27,28,29	0.848	0.35	0.051	1.325	0.149	0.024
	Pintung	PD	22.13°N 120.88°E	16	2	H12,13	0.325	0.04	0.200	1.738	0.162	0.309
	Taitung	TD	22.83°N 121.18°E	5	2	H12,17	0.600	0.05	1.224	0.626	0.300	0.090
Penghu Archipelago (Lineage B)				33	10		0.686	0.18	−1.296	−2.383 *	0.067	0.038
	Penghu	PH	22.53°N 119.62°E	14	7	H1,2,18,22,24,25,26	0.692	0.19	−1.237	−1.557	0.089	0.585 *
	Qimei	CM	23.22°N 119.44°E	13	4	H1,2,16,22	0.603	0.12	−0.754	0.438	0.157	0.484 *
	Wangan	WA	23.34°N 119.49°E	6	5	H1,2,21,22,23	0.933	0.26	−0.399	−1.121	0.167	0.076
Coast of mainland China (Lineage C)				23	10		0.771	0.13	−1.876 *	−5.060 *	0.076	0.003
	Kinmen	KM	24.45°N 118.38°E	12	8	H4,5,6,7,8,9,10,11	0.848	0.17	−1.942 *	−3.762 *	0.093	0.004
	Matsu	MZ	26.21°N 119.98°E	11	5	H3,4,7,9,14	0.709	0.09	−1.219	−1.684	0.135	0.002
Ryukyu (Lineage D)				13	2		0.154	0.01	−1.149	−0.537	0.266	0.023
	Ishigaki	IS	24.36°N 124.11°E	13	2	H15,30	0.154	0.01	−1.149	−0.537	0.266	0.023
Total				117	30		0.899	0.46	−1.128	01506.888 **	0.056	0.020 *

*N*, Number of individuals sampled; *N*h, number of haplotypes; *h*, haplotype diversity; π, nucleotide diversity; *R*_2_, Ramos-Onsins and Rozas’ *R*_2_ test; SSD, sum of squared differences of the mismatch distribution (significance as indicated).

* *p* < 0.05;

** *p* < 0.01.

**Table 2:** AMOVA results for testing genetic subdivision between populations using mtDNA for four groups. (1) Taiwan Island group (populations TP, WW, PD and TD); (2) Panghu Archipelago group (populations PH, CM and WA); (3) Coast of mainland China group (populations KM and MZ); and (4) Ryukyu group (population IS).

Source of variation	Variance components	Percentage of variation
Among geographic districts	2.603 ***	71.31
Among populations within geographic district	0.127 **	3.49
Within populations	0.92 ***	25.2

** *p* < 0.01,

*** *p* < 0.001.

**Table 3:** Results of SAMOVA tests for increasing K (number of groups) values. Asterisks (^*^) denote values with *p* < 0.05 based on 100 simulations. See Table 1 for the locality codes.

Number of groups	Groupings	Φ_CT_ %	Variance among groups
2	(TP, WW, PD, TD, PH, CM, WA, KM, MZ) (IS)	0.346	34.61
3	(TP, WW, PD, TD) (PH, CM, WA, IS) (KM, MZ)	0.533	53.28
4	(TP, WW, PD, TD) (PH, CM, WA) (KM, MZ) (IS)	0.713 ^*^	71.31
5	(TP, WW) (PD, TD) (PH, CM, WA) (KM, MZ) (IS)	0.699 ^*^	69.86
6	(TP, WW) (PD, TD) (PH, CM) (WA) (KM, MZ) (IS)	0.698 ^*^	69.81
7	(TP, WW) (PD) (TD) (PH, CM, WA) (KM) (MZ) (IS)	0.671 ^*^	67.06

**Table 4:** Point estimates and 95% confidence intervals for joint estimates of θ and migration (*N*m) in *Lunella granulata* from MIGRATE.

Lineage	θ	Taiwan	Ryukyu	China	Penghu
Taiwan	0.0058 (0.0045, 0.0077)	-	0	0	0.761 (0.14, 2.68)
Ryukyu	0.0059 (0.0037, 0.0101)	0	-	0	0.765 (0.114, 3.474)
China	0.0064 (0.0045, 0.0093)	0	0.297 (0.022, 1.574)	-	0
Penghu	0.0084 (0.0066, 0.011)	0	0	0	-
